# No Evidence of Presence of Parvovirus 4 in a Swedish Cohort of Severely Immunocompromised Children and Adults

**DOI:** 10.1371/journal.pone.0046430

**Published:** 2012-09-26

**Authors:** Thomas Tolfvenstam, Oscar Norbeck, Lars Öhrmalm

**Affiliations:** Department of Medicine, Solna, Infectious Disease Unit, Center for Molecular Medicine, Karolinska Institutet, Karolinska University Hospital, Stockholm, Sweden; University Hospital San Giovanni Battista di Torino, Italy

## Abstract

The recently discovered human parvovirus 4 (PARV4) has been associated with seropositivity for human immunodeficiency virus, hepatitis B virus and hepatitis C virus. High prevalence is seen especially in intravenous drug users. The virus has been detected in blood products and persons who have been repeatedly transfused have shown to be a risk-group. Furthermore, reports from different parts of the world suggesting a prevalence ranging from zero to one third of the healthy population and the virus is thought to cause a latent or persistent infection. We investigated the presence of PARV4 DNA and parvovirus B19 (B19) DNA in serum from 231 severely immunocompromised cancer patients that have been exposed for blood products. Compared to B19, which was found in 3.9% of the patients, we found no evidence of PARV4. Our results may indicate a very low prevalence of the virus in Sweden, and it would be useful to measure the real PARV4 exposure of the healthy population as well as individuals with known risk factors by serology.

## Introduction

Parvovirus 4 (PARV4) genotype 1 was discovered seven years ago [1] and subsequently genotypes 2 and 3 were described in 2006 and 2008, respectively [2], [3]. PARV4 has been associated with seropositivity for human immunodeficiency virus (HIV), hepatitis B virus (HBV) and hepatitis C virus (HCV) [4]–[12] and high prevalence is seen especially in intravenous drug users [4], [6], [7], [9], [12]–[14]. The virus has been detected in blood products [3], [15]–[19], and persons with hemophilia who have been repeatedly transfused have shown to be a risk-group [9], [20]. However, in a recent study, PARV4 seropositivity was also associated with exposure to injections of medications other than blood products [21]. Thus, the virus has been considered to be transmitted primarily parenterally [9], [21], but high rates of seropositivity in low-risk individuals in Africa suggest that other pathways should exist [22], [23]. Furthermore, in a study from Taiwan, PARV4 DNA was detected in plasma of three mothers and their newborns with hydrops, indicating transmission via placenta [24]. This is also one of the few studies demonstrating a possible association between the virus and disease. The reported prevalence of the virus in healthy individuals from different parts of the world varies. In a recent study from China, PARV4 DNA was detected in 20% of serum samples derived from HCV and HBV seronegative healthy controls [5].Although one study of French blood donors showed detectable PARV4 DNA in 24% of the cases [25], other reports from Europe and also from USA show lower frequency (0–4%) in the healthy population [9], [19], [26]–[29]. Although PARV4 may not cause long-term viremia, viral DNA has been detected in tissues lifelong after exposure, suggesting latency or persistence [10], [11], [30].

In a cross-sectional study, we investigated the presence of PARV DNA in serum of severely immunocompromised children and adults with various cancer diagnoses. Because of frequent transfusions of blood products, we assumed these patients to be at high risk of acquiring PARV4 infection. Moreover, due to the immunosuppression they would be less able to control an acute, persistent, or latent infection as seen with parvovirus B19 (B19) [31].

## Materials and Methods

Adult patients were, after giving their informed written consent, eligible for enrollment. Pediatric patients were enrolled after informed oral consents from the children or their guardians which was documented by the informing physicians. The study and both consent procedures were approved by The Regional Ethical Review Board in Stockholm (www.epn.se). In response to a decline in hemoglobin concentration and/or platelet count, red cell and platelet concentrates, respectively, were administered. In a few cases of sepsis, fresh frozen plasma was used. Coagulation factors were not administered. All patients were sampled in a severely immunocompromised state indicated by an absolute neutrophil count ≤500/mm^3^ which predominantly was accompanied by severe or moderate lymphopenia. The seroprevalence of HIV, HBV, and HCV was extracted from the patients' medical records. Collected samples were stored at −80°C. Total viral nucleic acids were extracted with QIAamp MinElute Virus Spin Kit (QIAGEN, US) according to the manufacturer's protocol with 200 µL of specimens eluted into 50 µL of extract. A real-time PCR assay designed to amplify an 103 bp product of the region from the gene for VP1 on the three known PARV4 genotypes [1]–[3] (prototype isolate: AY622943) was performed on an ABI 7500 Real-Time PCR System (Applied Biosystems) and carried out in a total 50-µL reaction mixture including 25 µL of TaqMan Universal PCR Master Mix (Applied Biosystems) and 10 µL of template. The concentrations of forward primer (5′-GCG ATG AAG GAG CTG ATT CC-3′), reverse primer (5′-AGA GGA TTA CCA GGA CCA ACA TAA TT-3′), and probe (5′-Cy5-ACC AGA CCT TGA GCG GCC TGC C-BHQ3-3′) were optimized with plasmids of known concentrations, reaching a sensitivity of 3 copies/reaction. The cycle program was 50°C for 2 min → 95°C for 10 min → 40 cycles of 15 sec at 95°C and 60 sec at 60°C. The assay was able to detect PARV4 in a positive plasma sample generously provided by Dr Sally A. Baylis in Germany. As a reference, B19 DNA was detected by a real-time PCR assay previously described [31].

## Results

A total of 191 adults and 40 children were included in the study. Approximately half of the adults were treated for chronic or acute leukemia, one third for lymphomas, and the rest for myelomas. Two thirds of the children were undergoing treatment for a hematological malignancy and one third for solid tumors. PARV4 DNA could not be detected in any of the samples. However, B19 DNA was found in 7 adults (3.7%) and in 2 children (5.0%; table 1). All were low titers except from one adult with approximately 10,000 copies/mL peripheral blood. No one of the children was seropositive for HIV, HBV, or HCV, whereas in the adult cohort two were positive for HBV and one for HCV. No prior intravenous drug abuse was reported in the patients' medical records.

**Table 1:** Characteristics of the parvovirus B19 positive and negative patients.

	Gender	Age	PARV4 DNA	No. of positive serology
	(F/M)	Median (range)	(Pos/Neg)	(HBV/HCV/HIV)
*B19 positive*				
Adults, n = 7	5/4	64 (40–77)	0/7	0/0/0
Children, n = 2	1/1	5 (3–7)	0/2	0/0/0
*B19 negative*				
Adults, n = 184	77/107	60 (20–86)	0/184	2/1/0
Children, n = 38	22/16	6.5 (0.5–18)	0/38	0/0/0

NOTE. F, female; M, male; PARV4, parvovirus 4; Pos, positive; Neg, negative; HBV, hepatitis B virus; HCV, hepatitis C virus; HIV, human immunodeficiency virus; B19, parvovirus B19; n, number.

## Discussion

To our knowledge there are no reports on the prevalence of PARV4 in Sweden, and our findings may suggest a low prevalence. Except from being recipients of different blood products, our study cohorts included low-risk individuals regarding reported risk factors such as HIV, HBV, and HCV seropositivity and usage of intravenous drugs. However, there are reports on PARV4 DNA in the blood of the healthy population in our part of the world [19], [25], [27], [28], and the virus is suggested to cause latent or persistent infection [10], [11], [30]. We thus believed that the immunocompromised state of our patients would make it even more likely to detect PARV4 DNA in peripheral blood. This was seen in an Italian study of healthy controls and allogeneic transplant recipients [28]. In another study the prevalence of PARV4 DNA in hemodialysis patients and lung transplant patients was compared [32]. Although DNAemia was detected in 14% of the plasma samples from the lung transplant recipients, the prevalence was less than half compared to the hemodialysis cohort (31%). This difference could be explained by difference between the groups regarding seropositivity for HIV, HBV, and HCV, but the authors discuss, based on their earlier data on the healthy population [25], possible undiscovered aspects of PARV4 infection with respect to the immunosuppressive treatments. In the only study from northern Europe, Lahtinen et al found only one out of 115 healthy students in Finland being weakly IgM positive [4]. Larger studies are needed to show if the prevalence of PARV4 is low in the Nordic countries as indicated by these results.

We used serum samples for detection of PARV4 DNA by PCR. Although plasma has been used in most studies, PARV DNA has been detected in human serum before [5], [13], [28]. Furthermore, the PCR assay was able to amplify DNA from a positive control. B19 DNA was detected which indicate that the DNA should not have been lost in the extraction phase. The absence of PARV4 in our cohorts should thus not be due to the choice of specimen or assay.

In conclusion, we failed to observe a high prevalence of PARV4 DNA in these cohorts of highly transfused and severely immunocompromised patients. It would be useful to measure the real PARV4 exposure of the Swedish healthy population as well as individuals with known risk factors by serology.
